# Changes in contact patterns shape the dynamics of the COVID-19 outbreak in China

**DOI:** 10.1126/science.abb8001

**Published:** 2020-04-29

**Authors:** Juanjuan Zhang, Maria Litvinova, Yuxia Liang, Yan Wang, Wei Wang, Shanlu Zhao, Qianhui Wu, Stefano Merler, Cécile Viboud, Alessandro Vespignani, Marco Ajelli, Hongjie Yu

**Affiliations:** 1 School of Public Health, Fudan University, Key Laboratory of Public Health Safety, Ministry of Education, Shanghai, China.; 2 ISI Foundation, Turin, Italy.; 3 Hunan Provincial Center for Disease Control and Prevention, Changsha, China.; 4 Bruno Kessler Foundation, Trento, Italy.; 5 Division of International Epidemiology and Population Studies, Fogarty International Center, National Institutes of Health, Bethesda, MD, USA.; 6 Laboratory for the Modeling of Biological and Socio-technical Systems, Northeastern University, Boston, MA, USA.

## Abstract

Intense nonpharmaceutical interventions were put in place in China to stop transmission of the novel coronavirus disease 2019 (COVID-19). As transmission intensifies in other countries, the interplay between age, contact patterns, social distancing, susceptibility to infection, and COVID-19 dynamics remains unclear. To answer these questions, we analyze contact survey data for Wuhan and Shanghai before and during the outbreak and contact-tracing information from Hunan province. Daily contacts were reduced seven- to eightfold during the COVID-19 social distancing period, with most interactions restricted to the household. We find that children 0 to 14 years of age are less susceptible to severe acute respiratory syndrome coronavirus 2 (SARS-CoV-2) infection than adults 15 to 64 years of age (odds ratio 0.34, 95% confidence interval 0.24 to 0.49), whereas individuals more than 65 years of age are more susceptible to infection (odds ratio 1.47, 95% confidence interval 1.12 to 1.92). Based on these data, we built a transmission model to study the impact of social distancing and school closure on transmission. We find that social distancing alone, as implemented in China during the outbreak, is sufficient to control COVID-19. Although proactive school closures cannot interrupt transmission on their own, they can reduce peak incidence by 40 to 60% and delay the epidemic.

## Who and what next?

The coronavirus 2019 (COVID-19) pandemic has brought tighter restrictions on the daily lives of millions of people, but we do not yet understand what measures are the most effective. Zhang *et al.* modeled virus transmission in Wuhan, China, in February 2020, investigating the effects of interventions ranging from patient management to social isolation. Age-mixing patterns were estimated by contact surveys conducted in Wuhan and Shanghai at the beginning of February 2020. Once people reduced their average daily contacts from 14 to 20 down to 2, transmission rapidly fell below the epidemic threshold. The model also showed that preemptive school closures helped to reduce transmission, although alone they would not prevent a COVID-19 outbreak. Limiting human mixing to within households appeared to be the most effective measure.

*Science*, this issue p. 1481

## Teaser

Reducing social contacts on the scale achieved in Wuhan and Shanghai in early 2020 could be sufficient to bring a COVID-19 outbreak under control.

The novel coronavirus disease 2019 (COVID-19) epidemic caused by severe acute respiratory syndrome coronavirus 2 (SARS-CoV-2) began in Wuhan City, China, in December 2019 and quickly spread globally, with 2,063,161 cases reported in 185 countries or regions as of 16 April 2020 (*1*). A total of 82,692 cases of COVID-19, including 4632 deaths, have been reported in mainland China, including 50,333 cases in Wuhan City and 628 cases in Shanghai City (*2*). The epidemic in Wuhan and in the rest of China subsided after implementation of strict containment measures and movement restrictions, with recent cases originating from travel (*3*). However, key questions remain about the age profile of susceptibility to infection, how social distancing alters age-specific contact patterns, and how these factors interact to affect transmission. These questions are relevant to the choice of control policies for governments and policy-makers around the world. In this study, we evaluate changes in mixing patterns linked to social distancing by collecting contact data in the midst of the epidemic in Wuhan and Shanghai. We also estimate age differences in susceptibility to infection based on contact-tracing data gathered by the Hunan Provincial Center for Disease Control and Prevention (CDC), China. Based on these empirical data, we developed a mathematical disease transmission model to disentangle how transmission is affected by age differences in the biology of COVID-19 infection and altered mixing patterns owing to social distancing. Additionally, we project the impact of social distancing and school closure on COVID-19 transmission.

To estimate changes in age-mixing patterns associated with COVID-19 interventions, we performed contact surveys in two cities: Wuhan, the epicenter of the outbreak, and Shanghai, one of the largest and most densely populated cities in southeast China. Shanghai experienced extensive importation of COVID-19 cases from Wuhan as well as local transmission (*4*). The surveys were conducted from 1 February 2020 to 10 February 2020, as transmission of COVID-19 peaked across China and stringent interventions were put in place. Participants in Wuhan were asked to complete a questionnaire describing their contact behavior (*5*, *6*) on two different days: (i) a regular weekday between 24 December 2019 and 30 December 2019, before the COVID-19 outbreak was officially recognized by the Wuhan Municipal Health Commission (used as baseline); and (ii) the day before the interview (outbreak period). Participants in Shanghai were asked to complete the same questionnaire used for Wuhan but only report contacts for the outbreak period. For the baseline period in Shanghai, we relied on a survey conducted in 2017–2018 that followed the same design (*7*). In these surveys, a contact was defined as either a two-way conversation involving three or more words in the physical presence of another person or a direct physical contact (e.g., a handshake). Details are given in the supplementary materials (SM, sections 1 and 2).

We analyzed a total of 1245 contacts reported by 636 study participants in Wuhan and 1296 contacts reported by 557 participants in Shanghai. In Wuhan, the average daily number of contacts per participant was significantly reduced, from 14.6 for the baseline period (mean contacts weighted by age structure: 14.0) to 2.0 for the outbreak period (mean contacts weighted by age structure: 1.9) (*p* < 0.001). The reduction in contacts was significant for all stratifications by sex, age group, type of profession, and household size (Table 1). A larger reduction was observed in Shanghai, where the average daily number of contacts decreased from 18.8 (mean contacts weighted by age structure: 19.8) to 2.3 (mean contacts weighted by age structure: 2.1). Although an average individual in Shanghai reported more contacts than one in Wuhan on a regular weekday, this difference essentially disappeared during the COVID-19 outbreak period. A similar decrease in the number of contacts was found in the United Kingdom during the COVID-19 lockdown period (*8*).

**Table 1:** Number of contacts by demographic characteristics and location. *N* is the number of participants who provided non-missing contact data.

**Characteristics**	**Wuhan**					**Shanghai**				
	**Baseline period**		**COVID-19 outbreak**		**Difference^§^**	**Baseline period**		**COVID-19 outbreak**		**Difference^§^**
	***N*** **(%)^†^**	**Mean** **(95% CI^‡^)**	***N*** **(%)^†^**	**Mean** **(95% CI^‡^)**		***N*** **(%)**	**Mean** **(95% CI^‡^)**	***N*** **(%)**	**Mean** **(95% CI^‡^)**	
Overall	624 (100.0)	14.6 (12.9, 16.3)	627 (100.0)	2 (1.9, 2.1)	12.6***	965 (100.0)	18.8 (17.8, 19.8)	557 (100.0)	2.3 (2, 2.8)	16.4***
Sex										
Male	300 (48.1)	14.5 (12.2, 17.1)	301 (48)	1.8 (1.7, 2)	12.6***	474 (49.1)	19 (16.9, 21)	286 (51.3)	2.1 (1.9, 2.4)	16.9***
Female	324 (51.9)	14.7 (12.5, 17.1)	326 (52)	2.1 (2, 2.3)	12.5***	491 (50.9)	18.5 (16.8, 20.4)	271 (48.7)	2.6 (2.1, 3.6)	16***
Age group										
0–6 years	12 (1.9)	8.6 (3.4, 17.4)	12 (1.9)	2.2 (1.7, 2.8)	6.4***	88 (9.1)	11.6 (9.2, 14.3)	14 (2.5)	1.9 (1.7, 2.2)	9.7***
7–19 years	79 (12.7)	16.2 (12.7, 19.6)	79 (12.6)	2.1 (2, 2.2)	14.1***	141 (14.6)	27 (23.1, 30.7)	55 (9.9)	2.6 (2, 3.4)	24.5***
20–39 years	254 (40.7)	15.3 (12.8, 18)	256 (40.8)	2.1 (1.9, 2.2)	13.2***	236 (24.5)	22.4 (19.8, 25.9)	254 (45.6)	2.2 (2, 2.5)	20.2***
40–59 years	221 (35.4)	13.8 (11.4, 16.7)	220 (35.1)	2 (1.8, 2.2)	11.8***	233 (24.1)	19.9 (17.7, 23.3)	160 (28.7)	2.8 (2, 4.1)	17.1***
≥60 years	58 (9.3)	13.9 (7.9, 20.7)	60 (9.6)	1.4 (1.2, 1.7)	11.6***	267 (27.7)	12.6 (10.8, 14.7)	74 (13.3)	1.6 (1.3, 1.8)	11***
Type of profession										
Preschool	12 (1.9)	8.6 (3.4, 17.4)	12 (1.9)	2.2 (1.7, 2.8)	6.4***	79 (8.2)	10.4 (8, 13.3)	14 (2.5)	1.9 (1.7, 2.1)	8.5***
Student	107 (17.1)	14.6 (11.4, 18.2)	107 (17.1)	2.1 (2, 2.3)	12.5***	173 (17.9)	26.2 (23.1, 29.2)	71 (12.7)	2.5 (2, 3.4)	23.7***
Employed	391 (62.7)	15.4 (13.4, 17.4)	390 (62.2)	2.1 (1.9, 2.2)	13.2***	400 (41.5)	22.5 (20.7, 24.4)	354 (63.6)	2.5 (2.1, 3.2)	20***
Working-age not in the labor force	30 (4.8)	14.1 (5.7, 24.2)	31 (4.9)	1.8 (1.4, 2.4)	12.2***	29 (3)	14.5 (7.8, 24.2)	24 (4.3)	1.8 (1.3, 2.4)	12.6***
Retired	84 (13.5)	12.1 (7.2, 17.4)	87 (13.9)	1.5 (1.3, 1.7)	10.6***	278 (28.8)	11.8 (10.2, 13.2)	94 (16.9)	1.6 (1.3, 1.8)	10.2***
Household size										
1	45 (7.2)	10.5 (5.3, 17.2)	45 (7.2)	0.6 (0.1, 1.5)	9.9***	35 (3.6)	15.2 (10.1, 21.1)	61 (11)	0.3 (0.1, 0.5)	14.9***
2	73 (11.7)	12.6 (8.2, 18.3)	76 (12.1)	1.1 (1, 1.2)	11.5***	244 (25.3)	14.5 (12.7, 16.7)	138 (24.8)	1.4 (1.1, 1.7)	13.1***
3	282 (45.2)	14.8 (12.8, 17.3)	283 (45.1)	1.9 (1.8, 2)	13***	432 (44.8)	20.3 (17.7, 22.4)	216 (38.8)	2.2 (2, 2.3)	18.1***
4	133 (21.3)	11.9 (9.3, 15)	132 (21.1)	2.3 (2.2, 2.5)	9.6***	117 (12.1)	20.3 (16.5, 23.8)	78 (14)	3 (2.8, 3.3)	17.3***
≥5	91 (14.6)	21.5 (16.2, 27.3)	91 (14.5)	3.2 (2.9, 3.4)	17.8***	137 (14.2)	21.4 (18.2, 27)	64 (11.5)	5.9 (4, 9.9)	15.5***

†Can differ from total sample size (*N* = 636) because it also includes participants who had not recorded contacts during the baseline period or during the COVID-19 outbreak. Note that reduced denominators indicate missing data. Percentages may not total 100 because of rounding.

‡The 95% CIs on the mean are calculated by bootstrap sampling.

§Difference is calculated by the subtraction of the number of contacts during the outbreak from the number of contacts during the baseline period. *p* values are taken from a negative binomial regression with a single binary variable distinguishing the baseline period from the outbreak.

*p < 0.05; **p < 0.01; ***p < 0.001.

**Fig. 1:**
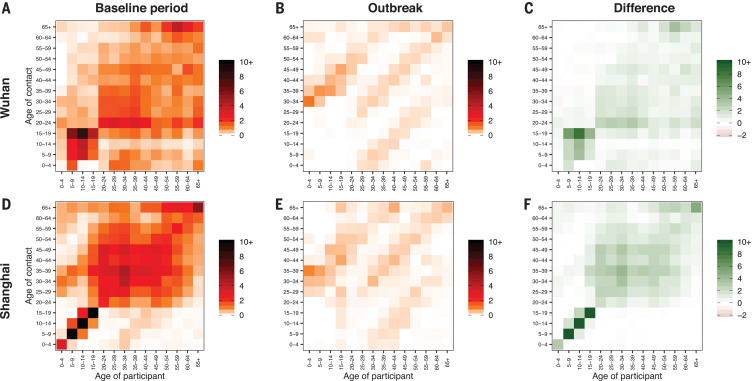
Contact matrices by age. (**A**) Baseline period contact matrix for Wuhan (regular weekday only). Each cell of the matrix represents the mean number of contacts that an individual in a given age group has with other individuals, stratified by age groups. The color intensity represents the number of contacts. To construct the matrix, we performed bootstrap sampling with replacement of survey participants weighted by the age distribution of the actual population of Wuhan. Every cell of the matrix represents an average over 100 bootstrapped realizations. (**B**) Same as (A), but for the outbreak contact matrix for Wuhan. (**C**) Difference between the baseline period contact matrix and the outbreak contact matrix in Wuhan. (**D**) Same as (A), but for Shanghai. (**E** and** F**) Same as (B) and (C), but for Shanghai.

The typical features of age-mixing patterns (*6*, *7*) emerge in Wuhan and Shanghai when we consider the baseline period (Fig. 1, A and D). These features can be illustrated in the form of age-stratified contact matrices (provided as ready-to-use tables in the SM, section 3.6), where each cell represents the average number of contacts that an individual has with other individuals, stratified by age groups. The bottom left corner of the matrix, corresponding to contacts between school-age children, is where the largest number of contacts is recorded. The contribution of contacts in the workplace is visible in the central part of the matrix, and the three diagonals (from bottom left to top right) represent contacts between household members. By contrast, for the outbreak period when strict social distancing policies were in place, many of the above-mentioned features disappear, essentially leaving the sole contribution of household mixing (Fig. 1, B and E). In particular, assortative contacts between school-age individuals are fully removed, as illustrated by differencing baseline and outbreak matrices (Fig. 1, C and F). Overall, contacts during the outbreak mostly occurred at home with household members (94.1% in Wuhan and 78.5% in Shanghai). Thus, the outbreak contact matrix nearly coincides with the within-household contact matrix in both study sites, and the pattern of assortativity by age observed for regular days almost entirely disappears (SM, section 3.6). These findings are consistent with trends in within-city mobility data, which indicate an 86.9% drop in Wuhan and 74.5% drop in Shanghai between early January and early February (see SM, section 4). Such a large decrease in internal mobility is consistent with most of the contacts occurring in the household during the outbreak period. Of note, the strict social distancing measures implemented in Wuhan and Shanghai did not entirely zero out contacts in the workplace, because essential workers continued to perform their activities (as observed in our data; see SM, section 3.5).

The estimated mixing patterns are based on self-reported contacts that can thus be affected by various biases. In particular, reported contacts for the baseline period in Wuhan may be prone to recall bias because contacts were assessed retrospectively. Further, because of the retrospective nature of the baseline survey in Wuhan, we were unable to account for the lower number of contacts during weekends. The more complete data from Shanghai did not suffer recall bias and allowed us to weight contacts for weekdays and weekends; sensitivity analyses suggest that this has little impact on results (SM, section 8.3). Another possible bias is that survey participants may have felt pressure to minimize reported contacts that occurred during the outbreak, given that social distancing was in place and strictly enforced by the government, even if the anonymity and confidentiality of the survey were emphasized. However, results are robust to inflating reported contacts outside of the home severalfold, suggesting that these compliance and social acceptability biases linked to the outbreak period do not affect our main findings (SM, section 8.2). Another caveat is that in parallel to population-level social distancing measures, case-based interventions were implemented and could have affected contacts, including rapid isolation of confirmed and suspected cases and quarantine of close contacts for 14 days. However, only a small portion of the population in the two study sites was affected by contact tracing and quarantine, thus having little to no effect on average contact patterns in the general population.

Next, to understand the interplay between social distancing interventions, changes in human mixing patterns, and outbreak dynamics, we need to consider potential age differences in susceptibility to infection. This is currently a topic of debate, because little information on the age profile of asymptomatic cases is available (*9*, *10*). To this aim, we analyzed COVID-19 contact-tracing information gleaned from detailed epidemiological field investigations conducted by the Hunan CDC (SM, section 5). Briefly, all close contacts of COVID-19 cases reported in Hunan province were placed under medical observation for 14 days and were tested using real-time reverse transcription polymerase chain reaction (RT-PCR). Those who tested positive were considered as SARS-CoV-2 infections. We estimated the odds ratios (ORs) for a contact of a certain age group to be infected, relative to a reference age group. We performed generalized linear mixed model regression to account for clustering and potential correlation structure of contacts exposed to the same index case (e.g., in the household). We included the age group and gender of a contact, type of contact, and whether the contact traveled to Hubei or Wuhan as regression covariates (SM, section 5). We found that susceptibility to SARS-CoV-2 infection increased with age. Young individuals (aged 0 to 14 years) had a lower risk of infection than individuals aged 15 to 64 years {OR = 0.34 [95% confidence interval (CI): 0.24 to 0.49], *p* < 0.0001}. By contrast, older individuals aged 65 years and older had a higher risk of infection than adults aged 15 to 64 years [OR = 1.47 (95% CI: 1.12 to 1.92), *p* = 0.005]. These findings are in contrast with a previous study in Shenzhen, where susceptibility to infection did not change with age (*9*).

Next, we explore how our data can inform control strategies for COVID-19. A key parameter regulating the dynamics of an epidemic is the basic reproduction number (*R*_0_), which corresponds to the average number of secondary cases generated by an index case in a fully susceptible population. We estimated the impact of interventions on *R*_0_, relying on our age-specific estimates of susceptibility to infection and contact patterns before and during interventions. We used the next-generation matrix approach to quantify changes in *R*_0_ (*11*) (SM, section 6). Additionally, to illustrate the impact of age-mixing patterns on the dynamics of the epidemic, we developed a simple SIR model of SARS-CoV-2 transmission (SM, section 6). In the model, the population is divided into three epidemiological categories: susceptible, infectious, and removed (either recovered or deceased individuals), stratified by 14 age groups. Susceptible individuals can become infectious after contact with an infectious individual according to the estimated age-specific susceptibility to infection. The rate at which contacts occur is determined by the estimated mixing patterns of each age group. The mean time interval between two consecutive generations of cases was taken to be 5.1 days, assuming it aligns with the mean of the serial interval reported by Zhang *et al*. (*3*).

**Fig. 2:**
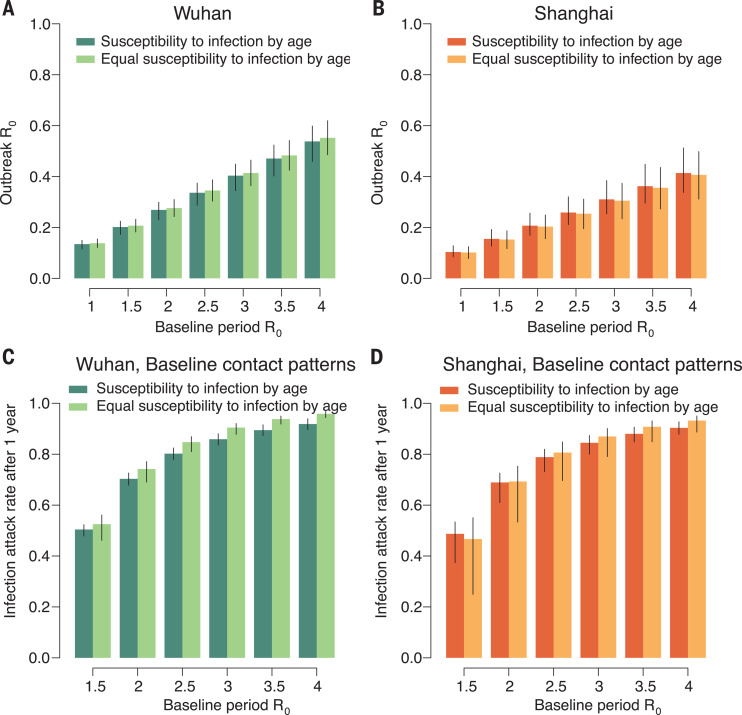
Effect of contact patterns on the epidemic spread. (**A**) Estimated *R*_0_ during the outbreak (mean and 95% CI), as a function of baseline *R*_0_ (i.e., that derived by using the contact matrix estimated for the baseline period). The figure refers to Wuhan and includes both the scenario accounting for the estimated susceptibility to infection by age and the scenario where we assume that all individuals are equally susceptible to infection. The distribution of the transmission rate is estimated through the next-generation matrix approach by using 100 bootstrapped contact matrices for the baseline period to obtain the desired *R*_0_ values. We then use the estimated distribution of the transmission rate and the bootstrapped outbreak contact matrices to estimate *R*_0_ for the outbreak period. The 95% CIs account for the uncertainty on the distribution of the transmission rate, mixing patterns, and susceptibility to infection by age. (**B**) Same as (A), but for Shanghai. (**C**) Infection attack rate 1 year after the initial case of COVID-19 (mean and 95% CI) as a function of the baseline *R*_0._ The estimates are made by simulating the SIR transmission model (see SM) using the contact matrix for the baseline period and considering the estimated susceptibility to infection by age and assuming that all individuals are equally susceptible to infection. The 95% CIs account for the uncertainty on the mixing patterns and susceptibility to infection by age. (**D**) Same as (C), but for Shanghai.

In the early phases of COVID-19 spread in Wuhan, before interventions were put in place, *R*_0_ values were estimated to range between 2.0 and 3.5 (*12*–*18*). In this analysis, we extended this range from 1 to 4 for the baseline period (i.e., before interventions). We find that the considerable changes of mixing patterns observed in Wuhan and Shanghai during the social distancing period led to a drastic decrease in *R*_0_ (Fig. 2). When we consider contact matrices representing the outbreak period, keeping the same baseline disease transmissibility as in the preintervention period, the reproductive number drops well below the epidemic threshold in Wuhan (Fig. 2A) and Shanghai (Fig. 2B). This finding is robust to relaxing assumptions about age differences in susceptibility to infection; the epidemic is still well controlled if SARS-CoV-2 infection is assumed to be equally likely in all age groups (Fig. 2, A and B). We also performed sensitivity analyses regarding possible recall and compliance biases of self-reported contacts as well as the definition of contact (i.e., considering only contacts lasting more than 5 min). The results are consistent with those reported here (SM, section 8).

In an uncontrolled epidemic (without intervention measures, travel restrictions, or spontaneous behavioral responses of the population) and for *R*_0_ in the range of 2 to 3, we estimate the mean infection attack rate to be in the range 53 to 92% after a year of SARS-CoV-2 circulation, with slight variation between Wuhan (Fig. 2C) and Shanghai (Fig. 2D). These estimates should be considered as an upper bound of the infection attack rate because they are based on a compartmental model that does not account for high clustering of contacts (e.g., repeated contacts among household members). If we consider a scenario in which social distancing measures are implemented early on, as the new virus emerges, the estimated *R*_0_ remains under the epidemic threshold and thus the epidemic cannot take off in either location. Furthermore, we estimate that the magnitude of interventions implemented in Wuhan and Shanghai would have been enough to block transmission for an *R*_0_ before the interventions of up to ~6 in Wuhan and ~7.8 in Shanghai.

**Fig. 3:**
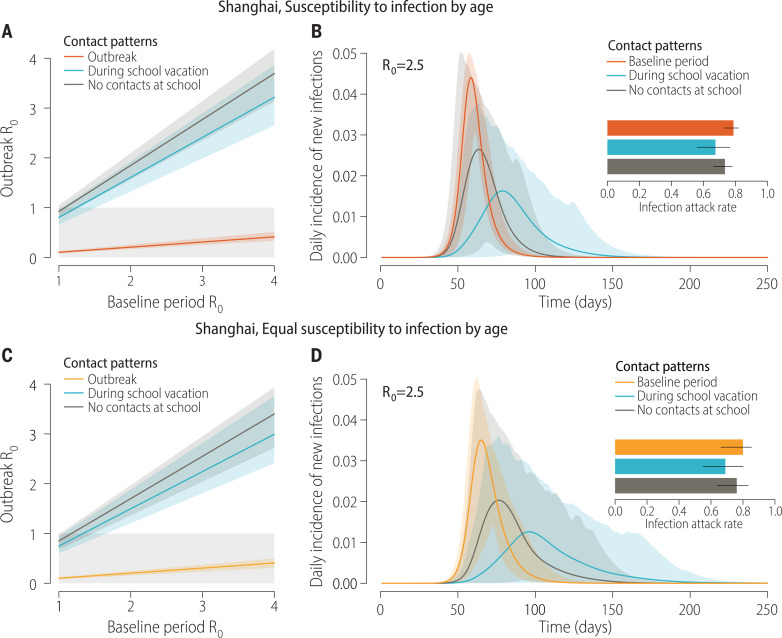
Effect of limiting school contacts on the epidemic spread. (**A**) Estimated *R*_0_ during the outbreak (mean and 95% CI), as a function of baseline *R*_0_ (i.e., that derived by using the contact matrix estimated for the baseline period). The figure refers to Shanghai and the scenario accounting for the estimated susceptibility to infection by age. Three contact patterns are considered: (i) as estimated during the COVID-19 outbreak, (ii) as estimated during school vacations (*7*), and (iii) as estimated for the baseline period, but suppressing all contacts at school. (**B**) Daily incidence of new SARS-CoV-2 infections (mean and 95% CI), as estimated by the SIR model, assuming age-specific susceptibility to infection (see SM). Three mixing patterns are considered: (i) as estimated for the baseline period, (ii) as estimated during school vacations (*7*), and (iii) as estimated for the baseline period, but suppressing all contacts at school. The inset shows the infection attack rate 1 year after the introduction of the first COVID-19 case (mean and 95% CI). (**C**) Same as (A), but assuming equal susceptibility to infection by age. (**D**) Same as (B), but assuming equal susceptibility to infection by age.

Next, we use the model to estimate the impact of preemptive mass school closure. We considered two different contact pattern scenarios, based on data from Shanghai: contacts estimated during vacation periods (*7*) and contacts estimated during regular weekdays, after all contacts occurring in school settings have been removed (*7*). Both scenarios represent a simplification of a school closure strategy. Indeed, school closures in response to the COVID-19 pandemic in China have entailed interruption of all educational on-site services. However, mixing patterns measured during school vacations indicate that a fraction of children still attend additional educational activities, as is typical in Chinese cities. On the other hand, when removing all contacts in the school setting, we do not consider potential trickle-down effects on the mixing patterns of other age groups; for instance, parents may need to leave work to take care of school-age children. Our modeling approach indicates that limiting contact patterns to those observed during vacations would interrupt transmission for baseline *R*_0_ up to 1.5 (Fig. 3, A and C). Removing all school contacts would do the same for baseline *R*_0_ up to 1.2. If we apply these interventions to a COVID-19 scenario, assuming a baseline *R*_0_ of 2 to 3.5, we can achieve a noticeable decrease in infection attack rate and peak incidence and a delay in the epidemic, but transmission is not interrupted (Fig. 3, B and D). For instance, for a baseline *R*_0_ of 2.5 and assuming a vacation mixing pattern, the mean peak daily incidence is reduced by about 64%. In the corresponding scenario where school contacts are removed, we estimate a reduction of about 42%. Overall, school-based closure policies are not sufficient to entirely prevent a COVID-19 outbreak, but they can affect disease dynamics and hence hospital surge capacity. It is important to stress that individuals aged 5 to 19 years in Shanghai represent 9.5% of the population (*19*), markedly lower than the mean in China [16.8% (*19*)] and other countries [including Western countries; e.g., 19.7% in the United States (*20*)].

The results of this study should be considered in light of the following limitations. In our simulation model, we estimated the effect of social distancing alone; combining social distancing with other interventions would have a synergistic effect to even further reduce transmission. It is likely that population-wide social distancing, case-based strategies, and decontamination efforts all contributed to achieve control in Wuhan and Shanghai, and their effect is difficult to separate out in retrospective observational studies. Our estimates of age differences in susceptibility to infection are based on active testing of 7375 contacts of 136 confirmed index cases. These data suffer from the usual difficulties inherent to the reconstruction of epidemiological links and detection of index cases. Contact data are useful, but seroepidemiology studies will be essential to fully resolve population susceptibility profiles to SARS-CoV-2 infection and disease. Although the age patterns of contacts were similar in the two study locations during the COVID-19 outbreak period, these patterns may not be fully representative of other locations in China and abroad, where social distancing measures may differ. Because reliable estimates of the contribution of asymptomatic SARS-CoV-2 infections to transmission are still lacking, we did not explicitly model differences between symptomatic and asymptomatic individuals. We considered a serial interval of 5.1 days (*3*), based on a prior estimate from China, at a time when case-based and contact-tracing intervention measures were in place, which tends to shorten the interval between successive cases. However, this choice does not affect the estimated changes in reproduction number between the baseline and outbreak periods. Modeling results may underestimate the effect of social distancing interventions because our results concentrate on the number of contacts and ignore the type of social interactions (e.g., increased distance between individuals while in contact or use of a face mask), which may have changed owing to increased awareness of the population (*21*, *22*). Finally, it is worth noting that our school closure simulations are not meant to formulate a full intervention strategy, which would require identification of epidemic triggers to initiate closures and evaluation of different durations of intervention (*6*). Nonetheless, our modeling exercise provides an indication of the possible impact of a nationwide preemptive strategy on the infection attack rate and peak incidence. To generalize these findings to other contexts, location-specific age-mixing patterns and population structures should be considered. Perhaps most importantly, strict lockdown strategies of the kind implemented in Wuhan, Shanghai, and other regions of the world are extremely disruptive economically and mentally, and more targeted approaches to block transmission are preferable in the long run. We do not necessarily endorse blunt lockdown policies here; we merely describe their impact on COVID-19 transmission based on the Chinese experience.

Our study provides evidence that the interventions put in place in Wuhan and Shanghai, and the resulting changes in human behavior, drastically decreased daily contacts, essentially reducing them to household interactions. This led to a dramatic reduction of SARS-CoV-2 transmission. As lockdown measures are put in place in other locations, human mixing patterns in the outbreak period could be captured by data on within-household contacts, which are available for several countries around the world (*5*–*7*, *23*–*25*). Moving forward, it will be particularly important to design targeted strategies for long-term control of COVID-19, including school- and work-based control strategies, along with large-scale testing and contact tracing (*26*–*28*). Research should concentrate on refining age-specific estimates of susceptibility to infection, disease, and infectiousness, which are instrumental to evaluating the impact of these strategies.

## Supplementary Materials

science.sciencemag.org/content/368/6498/1481/suppl/DC1 Materials and Methods Figs. S1 to S15 Tables S1 to S15 References (*30*–*41*) MDAR Reproducibility Checklist

## Appendix

View/request a protocol for this paper from *Bio-protocol*.
